# Cytogenotoxicity Evaluation of Young Adults Exposed to High Levels of Air Pollution in a Mexican Metropolitan Zone Using Buccal Micronucleus Cytome Assay

**DOI:** 10.1155/2021/6630861

**Published:** 2021-01-15

**Authors:** Ana Elizabeth González-Santiago, Guillermo Moisés Zúñiga-González, Belinda Claudia Gómez-Meda, Francisco Javier Gutiérrez-Corral, Ana Lourdes Zamora-Perez, María Guadalupe Sánchez-Parada

**Affiliations:** ^1^Departamento de Ciencias Biomédicas, División de Ciencias de la Salud, Centro Universitario de Tonalá, Universidad de Guadalajara, Tonalá, Jalisco, Mexico; ^2^Laboratorio de Mutagénesis, Centro de Investigación Biomédica de Occidente, Instituto Mexicano del Seguro Social, Guadalajara, Jalisco, Mexico; ^3^Instituto de Genética Humana Dr. Enrique Corona Rivera, Departamento de Biología Molecular y Genómica, Centro Universitario de Ciencias de la Salud, Universidad de Guadalajara, Guadalajara, Jalisco, Mexico; ^4^Instituto de Investigación en Odontología, Departamento de Clínicas Odontológicas Integrales, Centro Universitario de Ciencias de la Salud, Universidad de Guadalajara, Guadalajara, Jalisco, Mexico

## Abstract

Air pollution has become a serious public health problem globally. Recent studies support the harmful effect of air pollution on human health, in addition to scientific evidence that recognizes it as a human carcinogen. The buccal micronucleus cytome (BMC) assay is employed extensively to measure cytotoxic and genotoxic damage in a population exposed to environmental contamination. The objective of this study was to evaluate the cytotoxic and genotoxic effects in healthy young adults exposed to different levels of air pollution and to identify areas with air pollution rates above the regulatory limits. This study was performed through the BMC assay in oral mucosa samples from 80 healthy young adults from the Guadalajara metropolitan zone. Three highly contaminated areas were taken into account: Tlaquepaque, Miravalle, and Las Pintas. Las Aguilas, a less contaminated area, was used as a reference. The frequencies of nuclear abnormalities in the areas with the highest and lowest levels of air pollution were compared with the Mann–Whitney *U* test. In addition, an analysis of the concentration of environmental pollutants, particulate matter ≤ 10 *μ*m (PM10), ozone (O_3_), nitrogen dioxide (NO_2_), sulfur dioxide (SO_2_), and carbon monoxide (CO), were carried out in the mentioned areas, in order to identify the events above the regulatory limits in a year period. The results showed that young adults exposed to a higher concentration of pollutants showed higher frequencies of nuclear abnormalities. The individuals from the areas of Tlaquepaque, Miravalle, and Las Pintas showed cytotoxic damage since statistically significant differences were found in the abnormalities of pyknotic nuclei (PNs), condensed chromatin (CC), karyorrhexis (KX), and karyolysis (KL). The individuals who showed the most cytotoxic damage were from the Las Pintas area with higher frequencies in nuclear abnormalities (PNs, CC, KX, and KL) (*p* < 0.0001). Genotoxic damage was found in individuals from two zones, Miravalle and Las Pintas, with statistically significant differences in the abnormality of nuclear buds (NBUDs) (*p* < 0.0001). Our results suggest that exposure to high levels of air pollution in healthy young adults has an effect on cellular and nuclear integrity and thus in human health, since areas with higher air pollution showed an increase in cytotoxicity, specifically in early and late markers of cell death (CC, KX, PN, and KL) and genotoxic damage (BUDs).

## 1. Introduction

In the last decades, air pollution is one of the main public health problems and a cause of concern for the World Health Organization (WHO) due to an increase in industrialization and the use of automobiles [1].

According to the data of the Secretary of Environment and Territorial Development (SEMADET), Guadalajara City in Mexico, has positioned itself in recent years as one of the cities with the highest rate of air pollution and declarations of environmental contingencies due to an increase in population, number of vehicles, and industrialization [2].

Most of the air pollutants in large cities are generated by anthropogenic activities, such gasoline combustion from automobiles, along with pollution generated by industrial activities. Air pollutants are divided into two large groups: primary pollutants which are emitted directly into the atmosphere, where SO_2_, CO, and NO_2_ are recognized, and secondary pollutants, which are generated by photochemical processes over the primary ones, among which O_3_, H_2_SO_4_, HNO_3_, and others are included. Moreover, airborne pollutants include substances with no specified chemical composition, such as particulate matter (PM) and liquid and solid aerosols with a size of 0.001 to 10,000 microns. PM10 and PM 2.5 are relevant since they have effects on the respiratory system, generating an immune response and tissue damage [3]. The main contaminants according to the WHO guidelines are PM10, NO_2_, SO_2_, CO, and O_3_ [1]. All these pollutants are included in the air quality monitoring system of SEMADET Jalisco, Mexico [2].

The WHO recommended maximum mean levels for air pollutants that affect human health and increase mortality rates as follows: particulate matter PM10 (75 *μ*g/m^3^ 24-hour average), nitrogen dioxide (0.21 ppm 1-hour average), sulfur dioxide (0.11 ppm/24 hours), carbon monoxide (>11 ppm/8 hours), and ozone (>0.095 ppm/hour) [1].

Considerable toxicological evidence of the association of air pollution with human morbidity and mortality has been generated, since it is considered a predisposing mechanism for the development of chronic degenerative diseases, as well as deterioration in quality and life expectancy in urbanized areas. The detrimental effects of air pollution include alteration of the cardiovascular and respiratory systems and an increased risk of lung cancer and premature death [4–7]. Moreover, cytogenotoxic impact on people residing in communities exposed to high levels of air pollutants is documented in [8–11]. Harmful effect of environmental pollutants on the genetic material and cellular integrity is associated with the formation of free radicals and related reactive oxygen species, as well as its role in the genesis of apoptosis and carcinogenesis [12–15].

The buccal micronucleus cytome (BMC) assay has extended use in the measure of genotoxic and cytotoxic damage in human population exposed to air pollution [16–20], since micronuclei (MN) and other nuclear abnormalities such as binucleated cells (BNs), abnormally condensed chromatin (CC), karyorrhexis (KX), karyolysis (KL), pyknotic nuclei (PNs), and nuclear buds (NBUDs) are markers of DNA damage and cell death. Specifically, the nuclear abnormalities CC, KX, KL, and PNs are manifestations of cytotoxic effects, related to apoptotic and necrotic processes. Genotoxic effects generate MN and NBUD. BNs are generated by a defect in the mitotic spindle that leads to an aneugenic effect [21–24]. BMC test has the advantage of being minimally invasive and painless, since epithelial cells from a cheek swab are used. In addition, the sample represents cells from the first physical barrier of the respiratory tract [25–28].

The BMC assay assesses DNA damage and allows consideration to being a predictive biomarker, since an increase in nuclear abnormalities has been associated with genotoxicity and cancer cases [29–31]. Although the BMC assay has been utilized to evaluate genotoxicity and cytotoxicity in the Mexican population, currently, there are no studies that evaluate the effects of air pollution in healthy young adults. It is important to evaluate this population group, since air pollution harmful effects was assessed without other disease conditions that could contribute to cytogenotoxic effects. Furthermore, environmental pollution is a global priority issue, due to the relevance of exposure to pollutants on human health, climate change, and global warming [3, 32, 33].

In this study, the three most contaminated areas as well as one zone less contaminated as a reference, in the Guadalajara metropolitan area, were evaluated to assess the cytogenotoxic damage of the target population exposed to air pollution, in order to obtain evidence of its toxicological impact and demonstrate the need to establish intervention policies to reduce environmental impact.

## 2. Materials and Methods

### 2.1. Population and Study Area

**Figure 1:**
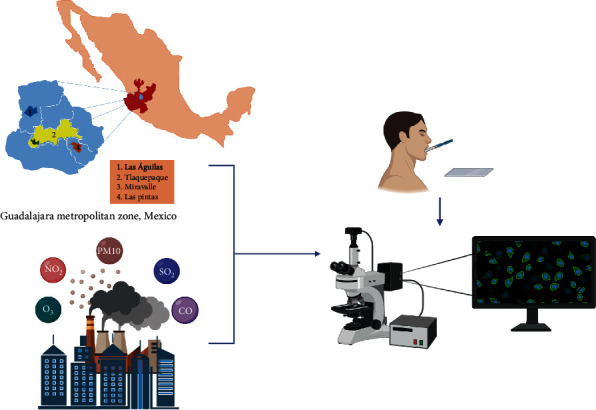
Geographical distribution of air monitoring stations in Guadalajara metropolitan zone, oral mucosal sampling, and BMC analysis.

This study was carried out in four areas of the Guadalajara metropolitan zone, three of them with high levels of air pollution (Tlaquepaque, Miravalle, and Las Pintas) and one more with low levels of pollution (Las Aguilas) (Figure 1). Each of these areas have fixed environmental monitoring stations.

The sampling was carried out in 2019 during the spring season (dry season), in which a total of 120 samples were taken, from which 20 samples were selected from each of the study areas. The final sample size was arbitrarily set at 20 participants for each zone (80 in total, 25 male and 55 female), for those who met the inclusion criteria. All participants were young adults with an average age of 18 (18.0 ± 0.28 years), recruited from public high schools. Before taking the sample, they signed an informed consent letter and a questionnaire was carried out, which included general information, as well as habits of alcoholism, smoking, drug addiction, exposure to solvents or fertilizers, chronic degenerative diseases, use of antioxidants, caffeine consumption, and eating habits. Samples from individuals with a history of alcoholism, smoking, drug addiction, and exposure to solvents and fertilizers were discarded. Similarly, those who had a history of consumption of vitamins C, D, and A, lutein, melatonin or commercial antioxidants before taking the sample (60 days before) or a history of excessive caffeine consumption (>150 mg/day) were also discarded, in addition to those who had a history of chronic disease. All the data obtained were handled under the criteria of ethics and confidentiality.

### 2.2. Pollutant Levels

The concentration of environmental pollutants was taken from the reports issued by the Ministry of the Environment and Territorial Development (SEMADET, 2019) during 2019 [2]. The environmental monitoring stations in the study areas have the capacity to measure the main atmospheric pollutants determined by the WHO as PM10, NO_2_, SO_2_, CO, and O_3_, and the monitoring stations are located 4.4 to 7.8 km apart from one another. These stations are part of the environmental monitoring network, strategically placed to measure the concentration of pollutants within the urban area.

To determine the environmental pollutants that were found above the permitted limits according to the SEMADET 2019 reports, and the areas with the greatest and least contamination, the permissible limit values for the concentration of pollutants of the Mexican official standards were taken as reference as in NOM-025-SSA1-2014 for PM10 levels [34], NOM-020-SSA1-2014 for O_3_ levels [35], NOM-021-SSA1-1993 for CO levels [36], NOM-023-SSA1-1993 for NO_2_ levels [37], and NOM-022-SSA1-2019 for SO_2_ levels [38], which are in accordance with the maximum of the average level recommended by the WHO [1, 39].

### 2.3. Sample Collection and Preparation and Cytogenetic Analysis

A confidential questionnaire was applied prior to taking samples to individuals who wished to voluntarily collaborate in the study, to later select the participants. Oral mucosa samples were collected from each of the individuals; they were asked to rinse their mouths with water to later carry out a direct scraping on both cheeks with a beveled slide. The sample was spread evenly on a coded slide, performing this procedure in duplicate on each participant. The samples were allowed to air dry to later be fixed in absolute ethanol for 48 h, and before their reading, they were stained with acridine orange, specific staining for nucleic acids.

Analysis of MN and other nuclear abnormalities (NBUDs, BNs, CC, KL, KX, and PNs) in the precoded samples was performed by a reader who blindly counted cells on an Olympus BX51 fluorescence microscope (Olympus, Tokyo, Japan) at 2000 cells/sample with a 100x immersion objective. Abnormalities were defined in accordance with established guidelines for nuclear morphology, staining pattern and intensity, and focal plane of the nucleus.

### 2.4. Statistical Analysis

The data of the nuclear abnormalities were expressed as mean ± standard deviation in 2000 cells. Statistical comparison of the means between the groups with the highest air pollution and the least air pollution was performed with the Mann–Whitney *U* test, to calculate the differences between each nuclear abnormality. The Statistical Package for the Social Sciences Software v.18.0 (SPSS, IBM Co., Armonk, NY, USA) was used for the analysis of the results. Statistical significance was considered with *p* values < 0.05.

### 2.5. Ethical Considerations

This study was approved by the Research Committee of the Tonalá University Center of the University of Guadalajara (CUTONALA/DCS/241/2018). It was carried out in accordance with institutional and government regulations, as well as in accordance with the guidelines of the Declaration of Helsinki. All participants signed an informed consent, and it was explained what their participation would consist of and the scope of the study.

## 3. Results and Discussion

### 3.1. Results

#### 3.1.1. General Characteristics

The frequency of damage and cytogenotoxic markers was evaluated in 80 young adults from four areas of the metropolitan area of Guadalajara, Jalisco, Mexico (20 individuals selected from each one), with different levels of atmospheric pollution, older than 18 years of both sexes, who signed prior informed consent. For their choice, it was considered that they lived in the margins established for each of the study communities (Las Águilas, Tlaquepaque, Miravalle, and Las Pintas) delimited by the locations of the monitoring station. Also, individuals that had more than a year in that residence, agreed to participate in the project and met the inclusion criteria.

#### 3.1.2. Nuclear Abnormalities by Specific Areas

The frequency of nuclear abnormalities in each area with the highest exposure to air pollution was compared with that in the area with the least exposure to air pollution (Table 1). The frequency of micronuclei and binucleated cells did not show significant differences when comparing the area with the lowest exposure to air pollution (Las Águilas) with each of the areas with the highest exposure to air pollution (Las Pintas, Miravalle, and Tlaquepaque). Pyknotic nuclei were significantly more frequent in the Las Pintas area with values of more than triple (*p* < 0.0001) concerning the group with less exposure. Condensed chromatins were more frequent in the areas with the highest exposure to atmospheric pollution (Las Pintas, Miravalle, and Tlaquepaque) compared to the area with the least exposure (Las Águilas). In the Las Pintas area, values up to four times higher were observed (*p* < 0.0001), the Miravalle area presented values three times higher (*p* < 0.0001), and Tlaquepaque presented with values two times higher (*p* < 0.001) compared to the area with less exposure. Karyorrhexis occurred more frequently in the three areas with the highest exposure to contamination, being the Las Pintas population the one that had the greatest significance with values twice higher compared to the area with the lowest exposure (*p* < 0.0001) followed by Miravalle (*p* < 0.0001) and Tlaquepaque (*p* < 0.01). Karyolysis showed results with a high frequency in the three areas with the highest exposure to air pollution, with values up to three times higher in the Las Pintas area compared to the area with the lowest exposure (*p* < 0.0001), followed by Miravalle (*p* < 0.001) and Tlaquepaque (*p* < 0.05). The NBUD showed significant differences in the majority of the areas with the highest exposure compared to those with the lowest exposure; in Las Pintas, the highest frequency was found (*p* < 0.0001) followed by Miravalle (*p* < 0.0001).

#### 3.1.3. Air Pollutant Concentrations in Areas of the Guadalajara Metropolitan Zone

The data on the concentration of environmental pollutants from the four study areas (Las Águilas, Tlaquepaque, Miravalle, and Las Pintas) of the Guadalajara metropolitan zone were reported by the environmental monitoring stations during 2019 and are shown in Table 2. Regarding the PM10 pollutant, events were found above the regulatory limits in the four study areas, being the Las Pintas area the one that presented the greatest number of events above the regulatory limits with a total of 105 during the year, followed by Tlaquepaque and Miravalle. The maximum concentration of this pollutant was also in the Las Pintas area.

In the same way, events were found above the regulatory limits in the four study areas of the ozone pollutant, being the Miravalle and Las Pintas zones which present the maximum values of the pollutant.

The area of Las Pintas was the only area that presented events above the regulatory limits for NO_2_, with a total of 77 throughout the year, in addition to presenting the maximum values of the pollutant. The rest of the pollutants did not show events above the regulatory limits according to the reported data.

### 3.2. Discussion

Guadalajara is a Mexican metropolitan zone located in the state of Jalisco. Approximately 5 million inhabitants live in the city, with a surface area of 151.6 km^2^ and a population density of 1622 inhabitants per km^2^. At present, 87.9% of the state's population lives in urban areas [40]. According to the World Urbanization Prospects 2018 Highlights, by the United Nations, Guadalajara is the 81st city in the world in terms of population [41]. From 1980 to 2019, the number of motor vehicles in Mexico has increased from about one million to more than 50 million in 2019. Jalisco ranked third nationwide with the highest number of registered motor vehicles in circulation, with 3,910,000 according to the National Institute of Statistic and Geography [42]. According to the United States Environmental Protection Agency (EPA), a typical passenger vehicle emits about 4.6 metric tons of carbon dioxide per year; thus, the total of vehicles in Guadalajara City could emit near 18 million metric tons of carbon dioxide into the atmosphere in a year period [43].

The Metropolitan Air Quality Index (IMECA) was established by Mexican authorities, first in Mexico City, and put into operation in 1977 [44]. The IMECA is constituted with information from an atmospheric monitoring network and includes data on ozone (O_3_), nitrogen dioxide (NO_2_), sulfur dioxide (SO_2_), carbon monoxide (CO), and the respirable fraction (PM10) of the total suspended particles [39]. Guadalajara City has an environmental monitoring network with 10 fixed stations; 8 stations have been functioning since 1996. Historically, 3 monitoring stations report out-of-norm peaks of air pollutants more frequently, which include Miravalle, Tlaquepaque, and Las Pintas. And the Las Águilas station has traditionally shown fewer peaks out of the norm in annual periods [2]. The generation of data from air quality monitoring stations around the world and their accessibility are essential to promote solutions to combat the regional and global effects of air pollution. In urban areas, air pollution exposure is considered a contributing mechanism for the development of chronic-degenerative diseases and quality of life deterioration [45]. Air pollution health risks include respiratory and cardiovascular diseases and are linked to premature death, as has been found in several studies [46–49].

Air pollutants such CO, NO_2_, and hydrocarbons are generated by vehicle exhaust emissions, while SO_2_ and suspended particulates are emitted mainly by industrial sources. Ozone is considered as a photochemical pollutant (secondary pollutant) and is formed from the atmospheric degradation of NMVOCs (Nonmethane Volatile Organic Compounds, which include benzene, xylene, propane, and butane; mainly emitted from transportation, industrial processes, and use of organic solvents) in the presence of NOx and sunlight. All of these pollutants are part of the air quality monitoring system [50]. The WHO Air Quality Guidelines provide values aimed at reaching adequate control emission politics and reductions on morbidity and mortality burdens (WHO, 2005). And also in this study, air pollutant regulatory limits were settled according to the official Mexican air quality standards for PM10 [34], O_3_ [35], CO [36], NO_2_ [37], and SO_2_ [38], respectively, as can be seen in Table 2.

In our study, contaminants outside the norm were found according to the Official Mexican air quality standards, especially for PM10, O_3_, and NO_2_. The results showed that young adults exposed to a higher concentration of pollutants have higher frequencies of nuclear abnormalities. The individuals from Tlaquepaque, Miravalle, and Las Pintas showed more cytotoxic damage with respect to Las Águilas, since statistically significant differences were found in the abnormalities of PNs, CC, KX, and KL. The individuals that showed the most cytotoxic damage were from the Las Pintas area with higher frequencies in nuclear abnormalities (Table 1). Genotoxic damage was found in individuals from two zones, Miravalle and Las Pintas, with statistically significant differences in the abnormality of NBUDs.

The increase in nuclear abnormalities assessed with the BMC assay has been associated with increased risk of accelerated aging, cancer, and neurodegenerative diseases [22]. The biomonitoring of cytotoxic and genotoxic damage is especially important for a city like Guadalajara, due to its high population density and high levels of environmental pollutants. When the nuclear anomalies associated with cytotoxicity (PNs, KX, CC, and KL) and those associated with genotoxic damage (NBUDs) were compared, highly significant differences (*p* < 0.0001) were found in the area of Las Pintas with respect to Las Aguilas. Moreover, the highest levels of PM10 and NO_2_ pollution and the highest number of days of the year above regulatory limits of the air quality standard were for the Las Pintas area.

The effect of PM10 particulate material on genotoxicity was studied in Mexico City by Calderón-Segura et al. [51]. They evaluate human cells exposed to extracts of airborne particles formed by polycyclic aromatic hydrocarbons (PAHs) in extractable organic matter adsorbed on PM10, according to distinct seasonal exposure. Bioactivated extracts with promutagens produced the highest rate of genotoxicity, while extracted organic material from PM10 without bioactivation was the least cytotoxic. According to the season of sampling, extracts are less cytotoxic in April and most cytotoxic in November. The results suggest that depending on the time of year, the composition of PM10 is different, and therefore, the toxicity is different during the prevalence of certain PM10 composition [51].

In a study carried out in Guadalajara City in 2017, a particle sampling of PM10 in the dry season identified species of anions and cations in the PM10 fraction by ion chromatography, such as F^−^, CH_3_COO^−^, Cl^−^, NO_2_^−^, Br^−^, NO_3_^−^, PO_4_^−^, SO_4_^2^^−^, C_2_O_4_^2^^−^, Li^+^, Na^+^, NH^4+^, K^+^, Ca^2+^, and Mg^2+^. The authors identified homogeneous concentrations of almost all individual species of anions and cations in the Guadalajara metropolitan zone. Also, the study suggests that PM10 composition is partly caused by secondary pollutants such as sulfate, nitrate, and ammonium, derived from direct gaseous precursors of combustion processes [52]. Interestingly, according to findings in an in vitro study carried out on human lung cells exposed to extracts of PM10 particulate material, the exposed cells developed genotoxicity and severe cytotoxic damage (pronecrotic changes). The genotoxic damage was rescued after administering a chelating agent, so it is suggested that this type of damage is associated with the presence of high levels of metal content in the particulate material PM10 [13].

To our knowledge, there are few studies evaluating the genotoxicity and cytotoxicity of particulate material (PM 2.5 and PM10) using the BMC technique. According to a study carried out in Brazil by De Oliveira Galvao et al., they assessed the occupational risk associated with artisanal cashew nut roasting in workers. Results showed biomarkers of genotoxicity (micronuclei and nuclear bud) and cytotoxicity (pyknosis, karyolysis, karyorrhexis, and condensed chromatin) higher in the exposed group compared with the controls [53]. The high concentrations of particulate matter and its composition appear to generate toxic effects in human cells, causing DNA damage and cell death [13, 51]. Our results show that in the Las Pintas area exist the necessary exposure conditions to PM10 to generate the genotoxic and cytotoxic damage that we found in the participants.

As previously reported, cytotoxic and genotoxic effects were found in the samples taken in Las Pintas. This zone reports NO_2_ levels 2.4 times higher than Mexican regulatory limits. The other atmospheric monitoring stations did not exceed the NO_2_ allowed values. In an ex vivo study of human nasal epithelial cells exposed to 0.1 ppm of NO_2_ for 0.5, 1, 2, and 3 hours, evaluated DNA fragmentation was assessed by comet assay and micronucleus induction. Genotoxic effects were demonstrated in short NO_2_ exposure durations (0.5 hours), a similar concentration found in urban areas. Micronucleus induction was reached only with prolonged exposure (3 hours) [14]. These results are congruent with the cytotoxicity and genotoxicity markers found for the Las Pintas zone. Although trends of greater exposure of pollutants in the air in the dry season can be found in the government official reports for several years (SEMADET) in the Guadalajara metropolitan zone, some data collected from monitoring stations during 2019 were not reported due to maintenance issues. It can be also considered that acute exposure (hours) to air pollutants is enough to induce cytogenotoxic damage.

Tlaquepaque and Miravalle were the other two areas with the highest number of events above regulatory limits of particulate pollutants (second and third places, respectively, of PM10 levels), as well as showing the highest events of ozone outside the norm (first and third places, respectively, but with maximum levels reported). It should be noted that ozone levels outside the norm were also found in the Las Aguilas station, but on fewer levels outside the regulatory limits with respect to other air quality monitoring stations.

Ozone can generate cytotoxic and genotoxic effects on cells of the human respiratory epithelium. According to a study carried out in a controlled environment chamber with ozone levels of 120 ppm (twice the value allowed for human health protection in EU legislation), ozone induced genotoxic damage in alveolar cells and less in fibroblasts. Ozone is a strong oxidant, and it decomposes producing reactive oxygen species (ROS) in aqueous solutions, including superoxide, hydroxyl radicals, and hydrogen peroxide. ROS could change the oxidant/antioxidant balance in the lung airway lining fluid leading to oxidative stress. Ozone-induced oxidative stress leads to several events in tissues and other cells, including activation and induction of inflammatory-related cellular responses such granulocytic inflammatory cells, activation of alveolar macrophages, damages to airway epithelial cells, and lung tissue dysfunction [54, 55].

Deleterious effects on human health linked to air pollutants depend on factors such as quantity and frequency introduced to the environment, diversity of the compounds, environmental conditions, toxicity, and reactivity. The reactivity of a primary atmospheric pollutant is important to consider, as they can react with other chemicals or generate secondary pollutants, as is the case for ozone [50, 56]. Exposure time is an important factor to consider since our results show the highest ozone peaks at the Miravalle area, but with fewer out-of-standard events than Tlaquepaque. However, we consider that there is a situation of health risk for the population due to the types of damage induced by ozone exposure, since we found out-of-norm levels higher than 0.1 ppm, according to the WHO safety guidelines, where it recommends a 1-hour average of 0.076 to 0.1 ppm ozone concentrations [1]. In contrast, in a study carried out on human lymphocytes exposed to ozone, differences in micronucleus frequency between pre- and postexposure numbers (of observed micronuclei per 1000 cells) were observed with ozone levels above 0.1 and 0.2 ppm. The authors also reported significant induction of apoptosis with ozone concentrations of 0.2 ppm. Another study in Mexico City reported an increase in DNA damage (by comet assay) in lymphocytes and nasal cells (but not buccal cells) in young adults in a polluted area of Mexico City in comparison with nonpolluted ones [57]. These authors found that the highest levels of DNA damage were found in nasal epithelial cells, since it is the first site of contact with ambient air, making these cells susceptible to genotoxic damage due to ozone exposure [57]. Therefore, we consider that it is necessary to carry out studies to evaluate the cytotoxicity and genotoxicity to the exposure of environmental pollutants, by collecting samples from both the nasal cells and the oral epithelium, to show if there are differences in toxicity.

Other epidemiological-molecular studies have highlighted the importance of using cytotoxicity and genotoxicity markers. In a two-year follow-up study in children, the authors sought to clarify the impact on the health of an Italian population with high rates of cancer in men. The frequency of micronucleated cells (MNC) in oral mucosa was evaluated to highlight the presence of alterations in the chromosomal structure and oxidative stress caused by exposure to air pollutants (IMP.AIR study). The register of environmental pollutants reports the maximum daily eight-hour mean value of ozone exceeded for 67 times the limit of 120 *μ*g/m^3^. The level of MNC detected in the IMP.AIR study appeared consistent with that reported during the winter in some Italian cities. However, many of the children participating in the study were found exposed to unhealthy factors concerning the domestic environment or lifestyles. These may have contributed to the occurrence of DNA alterations in children's exfoliated buccal cells, detectable through MNC [31]. In our study, we select individuals considering health and lifestyles, in order to reduce bias in the interpretation of the data.

Additionally, our results can be contrasted with two studies in which the buccal micronucleus cytome assay was used to determine cytogenetic damage abnormalities in children residing in urban areas [8, 9]. A study carried out in five Italian towns [9] showed maximum means for the markers of CC (29.10 ± 19.6), KX (13.83 ± 13.81), and KL (28.98 ± 18.76), also higher than those we found in Las Pintas (CC (8.05 ± 0.75), KX (4.90 ± 0.78), and KL (5.80 ± 0.76)), but also lower than those found in the other study conducted by Ceretti et al. [8] (CC (17.20 ± 6.13) and KX (7.44 ± 4.29)), except for KL (3.71 ± 3.37), which is lower than Las Pintas KL result. This variability could be explained by the differences that exist in the levels of contamination in the areas analyzed. We found a maximum mean of micronuclei (0.55 ± 0.51) in our study, near similar to that reported in the Villarini study (0.42 ± 0.54); however, when we compared MN with our population, statistically significant differences in MN were not found in any of the samples studied from each zone. It is possible that the levels of air pollution were not enough to increase levels of micronuclei, or as was the case that the damage to the genetic material was reflected by other parameters of apoptosis and cell death (CC, KX, PN, and KL) as we already previously discussed. In addition to an increase in nuclear BUDs, in comparison with the frequencies of BUDs reported by Villarini and Ceretti, our frequency is higher in the areas reported with greater contamination (Miravalle and Las Pintas). We had higher average of BUDs in Miravalle of 2.90 ± 0.71 and of 5.10 ± 0.85 in Las Pintas, while Villarini reports a maximum mean of 0.26 ± 0.48 and Ceretti reports 0.02 ± 0.04 [8, 9].

Air pollution is a sneaking agent that can induce damage on the young and old and is recognized for its anthropogenic origin. It is necessary to include programs to monitor the health of the population and should be integrated together with the politics about control of polluting emissions. The BMC assay is a noninvasive alternative to be integrated into a population health monitoring system in relation to assess cell levels of damage attributable to risk factors such as air environmental pollution.

## 4. Conclusions

Our results suggest that exposure to high levels of air pollution has an effect on cellular and nuclear integrity, since areas with higher air pollution showed an increase in cytotoxicity, specifically in early and late markers of cell death (CC, KX, PN, and KL) and genotoxic damage (BUDs), respectively. The individuals with greater cytogenotoxic damage were living in zones with pollutant concentration levels above the regulatory limit of PM10, NO_2_, and ozone. These results confirm previous findings that contribute to the confirmation of the cytotoxic and genotoxic effects of urban air pollution on human beings, and more efficient efforts are necessary to reduce the impact of air contaminants.

## Figures and Tables

**Table 1:** Frequency of micronuclei and other nuclear abnormalities in young adults exposed to air pollution in the Guadalajara metropolitan zone.

Nuclear abnormalities	Area with less exposure to air pollution	Areas with higher exposure to air pollution		
	Las Aguilas *n* = 20	Tlaquepaque *n* = 20	Miravalle *n* = 20	Las Pintas *n* = 20
MNs/2000 cells	0.50 ± 0.51	0.55 ± 0.51 NS	0.55 ± 0.51 NS	0.50 ± 0.60 NS
NBUDs/2000 cells	1.30 ± 0.97	1.10 ± 0.71 NS	2.90 ± 0.71 *p* < 0.0001	5.10 ± 0.85 *p* < 0.0001
BNs/2000 cells	0.70 ± 0.65	0.65 ± 0.67 NS	0.80 ± 0.61 NS	0.80 ± 0.52 NS
PNs/2000 cells	0.40 ± 0.59	0.75 ± 0.63 NS	0.55 ± 0.60 NS	1.35 ± 0.48 *p* < 0.0001
KX/2000 cells	1.80 ± 1.73	2.95 ± 0.82 *p* < 0.01	3.95 ± 0.82 *p* < 0.0001	4.90 ± 0.78 *p* < 0.0001
CC/2000 cells	1.85 ± 2.00	3.95 ± 0.82 *p* < 0.001	6.10 ± 0.85 *p* < 0.0001	8.05 ± 0.75 *p* < 0.0001
KL/2000 cells	1.75 ± 1.71	2.50 ± 0.51 *p* < 0.05	3.45 ± 0.60 *p* < 0.001	5.80 ± 0.76 *p* < 0.0001

The data for nuclear abnormalities are expressed as mean ± standard deviation. The comparison between the groups with the highest exposure to air pollution vs the group with the lowest exposure was made with the Mann–Whitney *U* statistical test. Statistical significance was considered with a value of *p* < 0.05. MNs: micronuclei; NBUDs: nuclear buds; BNs: binucleated cells; CC: abnormally condensed chromatin; KX: karyorrhexis; KL: karyolysis; PNs: pyknotic nuclei.

**Table 2:** Air pollutant concentrations in areas of the Guadalajara metropolitan zone and events above regulatory limits.

PM10 (*μ*g/m^3^) (24 hours)	Min. (*μ*g/m^3^)	Max. (*μ*g/m^3^)	Dispersion	Missing data *n* (%)	Events above regulatory limits in 2019 (>75 *μ*g/m^3^/24 hours)
Las Aguilas	10.70	80.28	0.3486	262 (71%)	1/365 days
Tlaquepaque	11.73	178.70	0.4976	93 (25%)	38/365 days
Miravalle	10.57	128.51	0.6693	216 (59%)	13/365 days
Las Pintas	10.1	260.56	0.6337	36 (9%)	105/365 days
Ozone (ppm/1 hour)	(ppm)	(ppm)			Events above regulatory limits in 2019 (>0.095 ppm/hour)
Las Aguilas	0	0.162	0.7371	351 (4%)	191/8760
Tlaquepaque	0	0.21	0.7412	240 (2%)	247/8760
Miravalle	0.001	0.225	0.6864	2201 (25%)	185/8760
Las Pintas	0.003	0.181	0.8137	1296 (14%)	9/8760
NO_2_ (ppm/1 hour)	(ppm)	(ppm)			Events above regulatory limits in 2019 (>0.21 ppm/hour)
Las Aguilas	0.001	0.029	0.6733	2128 (24%)	0/8760
Tlaquepaque	0	0.069	0.6791	6127 (69%)	0/8760
Miravalle	0	0.056	0.8048	1112 (12%)	0/8760
Las Pintas	0	0.518	1.4193	4738 (54%)	77/8760
CO (ppm/8 hours)	(ppm)	(ppm)			Events above regulatory limits in 2019 (>11 ppm/8 hours)
Las Aguilas	0.008	2.070	0.3727	17 (1%)	0/991
Tlaquepaque	0.142	6.96	0.5056	389 (39%)	0/991
Miravalle	0.006	2.00	0.4204	170 (17%)	0/991
Las Pintas	0.030	6.59	0.6796	153 (15%)	0/991
SO_2_ (ppm/24 hours)	(ppm)	(ppm)			Events above regulatory limits in 2019 (>0.04 ppm/24 hours)
Las Aguilas	0.0008	0.0036	0.2343	4 (1%)	0/365
Tlaquepaque	0.0024	0.0059	0.1815	65 (17%)	0/365
Miravalle	0.0004	0.0050	0.3592	43 (11%)	0/365
Las Pintas	0.0002	0.0102	0.3169	45 (12%)	0/365

The data as minimum and maximum levels detected for O_3_, NO_2_, CO, and SO_2_ are expressed as parts-per-million units (ppm), and PM10 levels are expressed as *μ*g/m^3^. Missing data is expressed as the number of data not reported or quantified by environmental monitoring stations per unit of time. Regulatory limits are settled according to the official Mexican air quality standards in NOM-025-SSA1-2014 for PM10 levels [34], NOM-020-SSA1-2014 for O_3_ levels [35], NOM-021-SSA1-1993 for CO levels [36], NOM-023-SSA1-1993 for NO_2_ levels [37], and NOM-022-SSA1-2019 for SO_2_ levels [38].

## Data Availability

The pollutant air level data used to support the findings of this study are freely available on the Air Quality Monitoring System from the Ministry of the Environment and Territorial Development (SEMADET) of the Mexican State of Jalisco, Mexico, from https://semadet.jalisco.gob.mx/medio-ambiente/calidad-del-aire.
